# Supplementary material for: Visceral Leishmaniasis in pregnancy and vertical transmission: A systematic literature review on the therapeutic orphans
Source: PLoS Negl Trop Dis. 2021 Aug 10;15(8):e0009650. doi: 10.1371/journal.pntd.0009650 (PMC8425569; doi:10.1371/journal.pntd.0009650)
Supplement: Attachment — Submitted filename: Response to reviewers comment.docx [file pntd.0009650.s006.docx]

The Editor,

PLOS Neglected Tropical Diseases

6^th^ July 2021

**Manuscript title: Visceral Leishmaniasis in pregnancy and vertical transmission: A systematic literature review on the therapeutic orphans**

Dear Editor,

We would like to thank you for the opportunity to revise and resubmit out review article. The reviewers’ comment were really useful and please find attached a revised version of the manuscript addressing the queries raised by the reviewers’ on a point-by-point basis. We believe the manuscript is much better for these changes and would like to thank the reviewers for a thorough review of our manuscript.

Yours sincerely,

Prabin Dahal

On behalf of the co-authors

**Reviewer #1: comments**

A comprehensive review on an understudied topic. It adds to the body of evidence for treatment of VL in pregnancy. I appreciate the suggestion in Box 1 "Proposed minimum variable recording and reporting for studies or case reports for VL in pregnancy" and also the comprehensive description of the reported cases on vertical transmission. In the discussion I miss interpretation of the results in the context of time and setting.

**Authors’ response:** We would like to thank the reviewer for the kind words. We have now added additional information on the geographical and time setting in the main text and discussion.

**Reviewer #1:** Line 93-95: This sentence is confusing. The ‘in utero’ congenital transmission is considered trans-placental. What is the supporting evidence for the congenital transmission through blood exchange during labour? According to Berger, 2017 (ref no. 10): “To date, the transplacental route is the only route that has been described for CT of Leishmania, and these studies represent ample proof of the capacity of Leishmania for placental invasion and CT via the transplacental route, based on demonstration of parasites in placental, fetal, and newborn tissue.”

**Authors’ response:** We have slightly revised the sentence and provided references to earlier publications that have discussed these possibilities as potential modes of vertical transmission.

<https://pubmed.ncbi.nlm.nih.gov/1536385/>

<https://pubmed.ncbi.nlm.nih.gov/2855677/>

<https://www.thelancet.com/journals/lancet/article/PIIS0140-6736(01)05214-X/fulltext>

The revised sentence now reads as (lines 110-115):

*“Further complexities arise from potential vertical transmission of the disease either congenitally (or through transplacental infection as a result of blood exchange during labour [17–19]. Such vertical transmission can induce in utero death or can be potentially deleterious to the foetus and infant [16,20,21]. While vertical transmission of VL is well-studied and established in animal studies, reports in humans are sporadic with observations of clinical manifestation several months post-partum [21–24]”*

**Reviewer #1:** Line 97-98: Such vertical transmission can induce in utero death or can be potentially deleterious to the foetus and infant. Consider moving this sentence before the previous sentence. Now you talk about reports for vertical transmission with a manifestation several months postpartum, and in the next sentence ‘such vertical transmission can cause intra uterine death’.

**Authors’ response:** The sentence is re-ordered.

**Reviewer #1:** Line 117: Typo   Expect = except

**Authors’ response:** Thank you. It is now corrected.

**Reviewer #1:** Line 127-132: What is expected to be the added value of this literature review? In the previous 2 reviews that are mentioned (Pagliano and Figuero, ref 9,22) it was already concluded that treatment of VL in pregnancy is imperative and that liposomal Amphotericin B is the drug of choice. Also in the WHO report on Control of the Leishmaniasis from 2010, liposomal Amphotericin B was already mentioned as the drug of choice for VL in pregnancy. However this review is more comprehensive and includes recent publications which makes it a valuable update of knowledge on VL in pregnancy.

**Authors’ response:** As the reviewer suggested, our review is a valuable update on the previous systematic reviews. The previous systematic review searched MEDLINE and Cochrane library. However, our review has searched a more comprehensive databases including the regional databases to identify relevant publications. The following databases were searched for our review: MEDLINE, Embase, Cochrane Databases, the World Health Organization Global Index Medicus: LILACS (Americas); IMSEAR (South-East Asia); IMEMR (Eastern Mediterranean); WPRIM (Western Pacific); ClinicalTrials.gov; and the WHO International Clinical Trials Registry Platform. Due to the rarity of the reports on VL during pregnancy, we also took a broad approach to include textbook, chapters, and conference abstracts in addition to the case reports and clinical studies identified by the previous systematic review. Therefore, our review remains the most comprehensive review on the topic to date and this has led to identification of several studies that were missed in the earlier systematic reviews. Thank you for the appreciation of our work.

**Reviewer #1:** Line 210: Typo Were 69 were

**Authors’ response:** It is now corrected.

**Reviewer #1:** Line 260: Ref 43 – splenectomy after birth? What was the indication for splenectomy? Suspected bleeding? Splenectomy is not a normal treatment intervention for VL.

**Authors’ response:** When the mother was treated with an antimony based regimen, there was persistence of parasitaemia when examined in the spleen sample and the authors described that splenectomy was performed.

It has now been added in the manuscript (now lines 303-305).
*“1 (1.1%) mother required splenectomy after delivery due to poor recovery and due to persistence of parasitaemia in the spleen sample [51].”*

**Reviewer #1:** Line 261: The baby who died at 2 months ago due to VL, is this not considered vertical transmission? This baby is not mentioned in line 262-263.

**Authors’ response:** We have explained this in the footnote of Table 3. The article doesn’t mention anything regarding the possibility of vertical transmission. Although it is likely to be a case of vertical transmission, in endemic areas, the possibility of differentiating a vertically acquired infection from an infection acquired through another mode remains difficult. Therefore, it was not explicitly considered (however, we have indicated in the table footnote for the readers).

The footnote appears in Table 3 (lines 401-402):

*“An article from Sudan (Adam 2009 [12]) described a case of a 2 months baby with parasites detected in lymph node. The article did not mention whether this could be a case of vertical transmission.”*

**Reviewer #1:** Line 360-366: Consider removing the calculation as it is probably far off?
**Authors’ response:** This has now been removed (also suggested by Reviewer 2).

**Reviewer #1:** Line 373: It would be informative to mention the (relative) disease burden per country. Some years back South Sudan had the second highest number of VL cases in the world after India, so it is not only a matter of local interest. Which countries have a high disease burden which is not represented in the available data?

**Authors’ response:** We agree that it is not only due to local interest. For example, in Sudan, which has one of the highest burden of VL, MSF has been keeping a robust database of treatments. Exact data regarding the total case burden for a given year is difficult to obtain. The Global Health Observatory maintained by the WHO provides the “reported” number of cases per country. Based on the WHO database, India, Sudan, Brazil, Ethiopia, Kenya, and South Sudan accounted for more than 80% of the total reported cases in 2019.

<https://www.who.int/data/gho/data/indicators/indicator-details/GHO/number-of-cases-of-visceral-leishmaniasis-reported>

The relative disease burden is now added in lines 86-88:

*“India, Sudan, Brazil, Ethiopia, Kenya, and South Sudan accounted for more than 80% of the total VL cases reported to the WHO in 2019 [3].”*

**Reviewer #1:** 373-384: There were different treatment regimens. What are the causes for this? The time frame of the included studies is very wide, so treatment regimens have changed over time. Increased availability of liposomal Amphotericin B over time must have played a role. As it is costly and it needs cold chain it may not be available in all resource-poor settings. What is considered the second best alternative in case Liposomal Amphotericin B is not available?

**Authors’ response:** The wide time span means that many drug regimens has been tested (as noted by the reviewer). Currently, Liposomal amphotericin B remains the safest regimen for the treatment in pregnancy based on the existing observational data. All other regimens are either contra-indicated for usage in pregnancy or are categorised into FDA class C or D (indicating evidence of risk for usage during pregnancy) (Table 1).

**Reviewer #1** 373-384: What is the role of the different strains of parasites in the different continents? The susceptibility to Amphotericin B is better in Asia than in Africa, I believe. I miss interpretation of the outcomes in the context of time and setting. (<https://www.ncbi.nlm.nih.gov/pmc/articles/PMC2889656/>) In zoonotic VL (the Mediterranean Basin, the Middle East, and Brazil) a total liposomal amphotericin B dose of ≥20 mg/kg is adequate to treat immunocompetent children and adults in these regions. The exact dosing schedule can be flexible (divided into doses of 10 mg/kg on 2 consecutive days or in smaller divided doses), but liposomal amphotericin B pharmacokinetics suggest that the initial dose will provide better tissue levels if at least 5 mg/kg is given. The schedule of 10 mg/kg/day on two consecutive days needs to be validated in adults.
For HIV-VL coinfection, Highly Active Antiretroviral Therapy should be a priority. There is an urgent need for multicenter trials of L-AmB as a first-line treatment and for secondary prophylaxis of VL in HIV-infected patients. In the anthroponotic cycle in the Horn of Africa, liposomal amphotericin B can be given at a total dose of 20 mg/kg however, a dose of 10–15 mg/kg may be adequate for South Asia.
In India, where a single infusion of L-AmB by itself at 7.5 or 5 mg/kg can induce cure rates of 90%–91%, combination regimen with lower doses of L-AmB with miltefosine or paromomycin is an option. With the preferential pricing, along with just one day of hospitalization, makes a single infusion of 10 mg/kg of L-AmB considerably less expensive and a viable option for the treatment of VL in the subcontinent.[53]. Well conducted trials of combination therapy with L-AmB is urgently needed.)

**Authors’ response:** As the reviewer remarked, Liposomal amphotericin B works well in Asia but has a lower efficacy in East Africa. The same parasite species is responsible for VL in Asia and Africa and hence the variation in susceptibility/efficacy of the drug is difficult to explain. The observed differences could likely be due to a combination of host, parasite, and drug factors. However, we do not have sufficient information if such could be the case for the treatment of VL during pregnancy and hence do not discuss the reasons for observation of such heterogeneity. We have now added the following sentences to highlight the differences in context (lines 428-431):

*“There was also geographical disparity in the treatment regimens used, reflecting either heterogeneity in treatment practices or the differential parasite susceptibility against drug regimens.*

**Reviewer #1:** Line 396: You identified a high risk of bias. What could this mean for the presented data? In other words do you expect over- or underestimation of cure rates/ mortality, what does it mean for the follow up data?

**Authors’ response:** We cannot clearly ascertain the impact of the bias on estimation of cure-rates or mortality based on the data available. It means that the quality of evidence obtained from the data is not optimal.

**Reviewer #1:** Did you find a risk factor for vertical transmission? E.g. in which trimester the mother was affected, duration / type of treatment.

**Authors’ response:** With the limited data available, we are not able to find any specific characteristics related to vertical transmission. However, we have provided case details for all the 26 cases of vertical transmission in Table 3.

**Reviewer #1:**For the checklist (Box 1) consider adding:   
Hb on admission,
Severity score (<https://journals.plos.org/plosntds/article?id=10.1371/journal.pntd.0005921>),
Need for blood transfusion during treatment,
Treatment response (clinical cure, parasitological cure)
Total dose of treatment given
Side effects
(Initial) Treatment outcome: Initial cure / non-response / defaulter / death
Ideally: Definite cure (at 6 months)
Delivery during treatment ? (abortion / stillbirth / premature birth / healthy birth)
Delivery complications (PPH?)
Ideally: Pregnancy outcome after completing treatment (abortion / stillbirth / premature birth / healthy birth)
Ideally: follow up of neonate born to mother treated during pregnancy (survival, signs of vertical transmission)

**Authors’ response:** Thank you for these suggestions. We have now incorporated these suggestions in Box 1.

**Reviewer #2: Comments**

**Reviewer #2:**

I appreciate the work of the authors on reviewing the literature about reported cases of VL in pregnant women. The determination of the safest VL treatments for mother-infant pairs, and the examination of cases of VL vertical transmission are valuable to physicians who typically relied on thin precedence to administer treatment. The synthesis of information about the treatment of VL in a vulnerable yet understudied population is meaningful. The comprehensive capture of cases from multiple literature sources and across 90 years is a strength of this review. However, I have highlighted below some concerns about insufficient context that should be addressed in the paper.

**Authors’ response:** We would like to thank the reviewer for the appreciation of our work and for several informative suggestions to improve our manuscript. We have made our best efforts to address the comments raised.

MAJOR COMMENTS

**Reviewer #2:**

I believe the authors will strengthen this review by adding a paragraph at the beginning of the introduction describing the signs, symptoms, and severity of VL. Is the disease short-term or long-term? Is it fatal? A quick search indicates a wide clinical spectrum including fever, weight loss, and severe anemia. This information is helpful to include because it provides more comprehensive context about VL’s burden.
**Authors’ response:** We have now added this information in the Introduction section of the manuscript (lines 89 to 100).

**Reviewer #2:**

I recommend adding a paragraph between the first and second paragraphs of the introduction (lines 85 and 86) about the typical approach to diagnosing and treating VL among non-pregnant women. The first lines of paragraph two indicate that diagnosis among pregnant women relies on symptoms and serology. Does diagnosis not typically involve symptoms and serology? Is splenic aspiration the typical approach to diagnosing VL?

**Authors’ response:** We have now added a new paragraph that discusses the diagnosis (lines 89 to 100).

**Reviewer #2:**

Line 88 mentions more severe anemia and increased requirements for blood transfusion among pregnant women. Is VL associated with anemia in the wider population? Does VL exacerbate existing anemia or is it associated with new-onset anemia? Are blood transfusions common among non-pregnant VL patients? Describing how treatment during pregnancy deviates from the norm brings to the foreground the particular risks of VL to mothers and infants.

**Authors’ response:**

It is well known that anaemia (severe anaemia) is one of the complications of VL. While we do not know the full extent of interaction between existing anaemia and the role of VL in its further exacerbation, several publications have described the requirements of blood transfusions either before the initiation of the treatment or during the post-treatment follow-up period. It has been recently shown that pregnant women with VL are at a 9-fold odds greater risk of blood transfusion than non-pregnant women with VL (See: <https://pubmed.ncbi.nlm.nih.gov/31978116/>). We would like to thank the reviewer for bringing these points. These have been added in the main text for further clarification.

We have now revised lines 102-106:

*“VL in pregnancy presents a number of challenges. During pregnancy, the use of splenic aspirate poses additional risk for the foetus. Pregnant women are also more likely to present with more severe anaemia than non-pregnant women and they are at an increased risk of requiring blood transfusion [11,12].”*

**Reviewer #2:**

I appreciate the inclusion of several types of published literature formats to capture as many cases as possible! Assessment of bias was helpful, but the authors would benefit from describing the classification of risk of bias for outcome assessment, adequacy of follow-up, and reporting of results. The authors mention that the risk of selection bias was high for single case reports and lower for cohort studies. This was very helpful. For outcome assessment, how did the authors ascertain low or high risk of bias? What duration of follow-up was classified as higher or lower risk bias? What made how a case was reported lead to a classification of higher/lower risk of bias? I recommend describing these parameters in the same way selection bias and ascertainment of exposure were described. Expand on the findings in the results section.

**Authors’ response:** Thank you for the suggestion**.** We have also provided additional description in the main text (lines 209-225).

Assessment of bias in “outcome ascertainment” is challenging as there are multiple outcomes being assessed during pregnancy – outcome of pregnancy, outcome of treatment response, assessment of congenital transmission. For the purpose of this review, pregnancy related outcomes were considered – as this is of direct consequence to the maternal and fetal health. Since these are objective assessment (eg: death, abortion, requirement of C-section etc), the outcome assessment domain was not considered on its own. Instead, the completeness of the reporting of outcome including reporting of treatment status was used for a composite assessment of “incomplete reporting” domain. In the interest of keeping the manuscript as succinct as we can, the exact details and reasons for classification of different bias status are presented in additional file: S1 Table. The methodological description in main text is kept to minimum.

**Reviewer #2:**

The authors did a good job of describing outcomes among the mothers and infants. However, the authors tended to mention important information such as dosing, treatment information, and latter outcomes briefly in parentheses instead of describing the cases in greater detail. For example, in line 252, the authors write, “… and for the third case, vertical transmission was suspected 8 months after birth (treated with sodium stibogluconate 20 mg/kg for 20 days and discharged)”.  This is one of only 3 cases of vertical transmission among those treated with L-AmB, so its circumstances merit commentary. A glance at the abstract indicates that the mother was co-infected with HIV. This is an extenuating circumstance that might complicate simple vertical transmission. Consider eliminating parentheses around important information, especially when describing specific or rare instances. See also Lines 250, 264-265, 271-272, 315-317, and 321

**Authors’ response:** Thank you for the suggestions. We have now added further descriptions of the cases and removed the parenthesis and provided full details instead.

**Reviewer #2:**

I recommend describing the time trends and geographic locations of the most common adverse birth outcomes to build a case for the dangers of a particular treatment. For example, 27% of women treated with pentavalent antimony experienced abortion or spontaneous abortion (line 258). Did all of these women live in the same country/continent? Were these abortions consistent over time, or were they more common a few decades ago? Did each woman receive the same (or similar) dose? It is clear that pentavalent antimony is associated with worse outcomes, but the context of these outcomes strengthens the evidence that the drug itself, and not alternative circumstances like delayed treatment, severe VL, or a lack of access to care, are to blame.

**Authors’ response:** Thank you for this suggestion. We have now added the time-period and geographical information for all the treatment regimens used through the manuscript, especially in the Results section. The clinical outcomes following L-AmB and Pentavalent antimony are also discussed with geographical and temporal aspects taken into consideration. These results are also presented in Table 4.

We have provided information regarding dose variability in the additional data file when they were reported (S2 Data).

**Reviewer #2:**

The results paragraph on untreated women (line 301) is the strongest in my opinion because it includes context about the extenuating circumstances of the reported adverse outcomes. I appreciate that the authors point out safety concerns, the rationale for a c-section, a lack of hospital resources, and suspected vertical transmission among the untreated women in this section. When possible, I recommend the authors include similar details about extenuating circumstances in other areas of the results section. Very interesting to read!

**Authors’ response:** We thank the reviewer for this appreciation.

**Reviewer #2:**

I believe the authors would strengthen the discussion by expanding on the possibility of time trends in treatment regimens. Given that studies of VL cases spanned 90 years, what changes have been observed over time?

**Authors’ response:** We have now added the time-period and geographical information for all the treatment regimens used. Although the review includes data from nearly 90 years, there were a substantially low number of publications prior to the 1990s. We have now added the temporal and geographical aspects in the results and discussion sections, where appropriate.

**Reviewer #2:**

The authors mention that prevalent antimony has been contraindicated in pregnancy (line 352). Is it known when this occurred? When did L-AmB become the “treatment of choice” (line 357)?

**Authors’ response:**

The sentence has been revised for clarity now regarding antimony. L-AmB has been used as the first line therapy for treatment against pregnancy in Kenya, Ethiopia, Somalia, Sudan, South Sudan, Uganda, and Brazil as outlined in the WHO policy document from 2010 and these are referenced in Table 1.

**Reviewer #2:**

The authors also mention that more than half of cases occurred after 2005 (line 372). Has the standard course of treatment changed in the past 16 years?

**Authors’ response:** We have provided information regarding dose variability in the additional data file when they were reported – it is difficult to discern any specific trend in the dosage adopted (S2 Data).

**Reviewer #2:**

The line of thinking in lines 360-370 is very confusing. It’s not clear where the 15,883 included patients come from (line 363) or how the authors estimated 96 cases per 100,000 people. Also, it may not be likely that everyone screened for eligibility had the disease, meaning 96 cases is likely an overestimate. The point taken is that cases of VL among pregnant women are underreported and recorded incidence is probably an underestimate. Since the example from IDDO records does not add to this conclusion, I recommend removing this paragraph for clarity.

Authors’ response: We appreciate the reviewer’s concern regarding the complexity associated with size estimation. These are very simplistic computations and to avoid further confusion, this has now been removed (also suggested by Reviewer 1).

**Reviewer #2:**

I strongly recommend adding a paragraph or two about geographic trends. The authors mention as an aside in line 371 that more than half of cases came from 2 countries. I believe that is a significant point that merits additional commentary. How might overrepresentation from Sudan and South Sudan impact results and conclusions? Is a certain treatment approach more common in a given region?

**Authors’ response:** We have now added the time-period and geographical information for all the treatment regimens used. Although the review includes data from nearly 90 years, there were a substantially low number of publications prior to the 1990s. We have now added the temporal and geographical aspects in the results and discussion sections, where appropriate.

**Reviewer #2:**

The inclusion of the checklist is a great idea!
Authors’ response: Thank you for the agreement.

**Reviewer #2:**

In line 393, I recommend clarifying that the limitations in reporting of VL in pregnancy correlate with limitations in the present work. The review is comprehensive but limited because it makes use of cases aside from peer-reviewed studies and included some studies translated through Google Translate. Conclusions are based on the over-representation of cases in Sudan and South Sudan, which may differ from treatment approaches in other nations.

**Authors’ response:** We have considered this in our limitations section and added lines 458-460:

*This finding is explained by the fact that this review included case reports and case series for comprehensiveness. Of the 72 articles included, 71 were case-reports or case-series and a single study was a retrospective cohort study with a comparator group.*

**Other comments:**

**Reviewer #2:** In lines 105-106, delays in treatment of PKDL do not appear to be relevant.
**Authors’ response:** It has been removed now.

**Reviewer #2:** For clarity, I recommend removing “(for example in miltefosine trials)” in line 112. Miltefosine is not formally introduced in the text until later, and the South Asian study referenced in line 115 isn’t a miltefosine trial. Consider including something along the lines of, “A study conducted in South Asia found that only one in every six doctors ruled out pregnancy before prescribing miltefosine, a medication which has been contraindicated in pregnancy.” 

**Authors’ response:** Thank you for this suggestion. We have revised accordingly.

**Reviewer #2:** It is unclear how Table 1 is organized. I recommend using alphabetical order for simplicity.
**Authors’ response:** Table 1 is organised based in order of publication year and we would like to keep it in the temporal order as it currently appears.

**Reviewer #2:** The meaning of lines 234-236 is unclear: “Ten were suspected of having carried the infection during their pregnancy, of whom 6 were cases of sub-clinical persistence of the parasites without the mother ever suffering from the disease previously.” Does this mean the parasites were present in women who were recently pregnant?

**Authors’ respon**se: These are cases that were identified retrospectively after the baby was found to have VL. These lines have been removed to avoid confusion.

**Reviewer #2:** I recommend clarifying that outcomes are presented by treatment type in line 240.
**Authors’ response:** The sentence is clarified as: *“The outcomes among 3 untreated mothers and 346 mothers who were received treated a treatment during pregnancy is presented by drug regimens”*

**Reviewer #2:** Although a bit redundant, readers would greatly benefit from transitions at the beginning of each treatment paragraph. It would be helpful if line 243 began with: “Among mothers treated with Liposomal Amphotericin (n=202)…”. Similarly, it would be helpful if line 255 began with: “Among mothers treated with pentavalent antimony (n=92)…” The authors do a good job providing context in lines 268 (amphotericin B deoxycholate), 279 (aminosidine), 290 (paromomycin), 294 (unclear), and 302 (untreated).

**Authors’ response:** Thank you for the suggestion. We have added this at the beginning of each paragraph.

**Reviewer #2:** For clarity, I recommend rephrasing lines 247-248. Consider something along the lines of, “There were five (2.9%) miscarriages, 1 (0.6%) stillbirth, and 1 (0.6%) premature birth. The mother’s trimester at the time of these outcomes was unclear. One fetal death was reported from a mother in her first trimester.”

**Authors’ response:** Thank you for this suggestion. We have revised as suggested.

**Reviewer #2:** In line 271, how was it determined that the mother was about 29 weeks pregnant? Was this the duration range of the mothers included in the referenced study?
**Authors’ response:** The exact time trimester for the mother who died was not available. Instead time into the pregnancy was available at group level for the case series of mothers who were described in the report.

**Reviewer #2:** In line 361, please spell out IDDO before the acronym.
**Authors’ response:** The acronym is already introduced in Methods section in line 162.

**Reviewer #2:** In lines 387-388, it is unclear why 6 months follow-up after treatment is insufficient.

**Authors’ response:** Of the 26 cases of vertical transmission, 12(46%) were identified after 6 months. This suggests that the standard follow-up period might be insufficient. The following sentence is now added: lines 447-449

“Our review identified 26 cases of vertically transmitted VL with a median time of detection of 6 months (range: 0–18 months) with 12(46%) of the cases identified after 6 months”

**Reviewer #3:**

Very useful systematic review. Under-researched topic. Unique body of evidence.
Large number of studies from a wide geographical area.

**Reviewer #3:** Your final sentence from the "Authors summary" is very relevant: our findings indicate that L-AmB should be the preferred treatment for VL
79 during pregnancy. " This could sentence could also fit in the abstract, where it appears less clearly (more cautiously): maybe too cautiously given the big difference in mortality% and miscarriage%.
This adds some evidence on the danger of using antimonials during pregnancy (despite the studies not being comparative and from different time periods).

Authors’ response: We have been cautious because of the limited data and several biases associated with the studies included. Therefore, we are not able to strongly recommend one regimen over the other.

**Reviewer #3:** ABSTRACT/Results part:
Outcomes were reported in 176 mothers treated (add "by") L-AmB with 4 (2.3%) reports of maternal deaths,...
Authors’ response: It has been corrected now.

**Reviewer #3:** Line 116-117: "Finally, there is a lack of active pregnancy registries for most of the antileishmanial drugs
117 expect for miltefosine." --> change expect to EXCEPT for...

Authors’ response: It has been corrected now.

**Reviewer #3:** Line 117:  I®mpavo (Profounda Inc.) --> change to ImpaVIDO

Authors’ response: It has been corrected now.
